# Nutrition Disturbances and Metabolic Complications in Kidney Transplant Recipients: Etiology, Methods of Assessment and Prevention—A Review

**DOI:** 10.3390/nu14234996

**Published:** 2022-11-24

**Authors:** Monika Górska, Ilona Kurnatowska

**Affiliations:** Department of Internal Medicine and Transplant Nephrology, Chair of Pulmonology, Rheumatology and Clinical Immunology, Medical University of Łódź, 90-153 Łódź, Poland

**Keywords:** kidney transplantation, nutrition disturbances, metabolic complications, dietary treatment

## Abstract

Nutrition disturbances occur at all stages of chronic kidney disease and progress with the decrease of the kidney filtration rate. Kidney transplantation (KTx) as the best form of kidney replacement therapy poses various nutritional challenges. Prior to transplantation, recipients often present with mild to advanced nutrition disturbances. A functioning allograft not only relieves uremia, acidosis, and electrolyte disturbances, but also resumes other kidney functions such as erythropoietin production and vitamin D3 metabolism. KTx recipients represent a whole spectrum of undernutrition and obesity. Since following transplantation, patients are relieved of most dietary restrictions and appetite disturbances; they resume old nutrition habits that result in weight gain. The immunosuppressive regimen often predisposes them to dyslipidemia, glucose intolerance, and hypertension. Moreover, most recipients present with chronic kidney graft disease at long-term follow-ups, usually in stages G2–G3T. Therefore, the nutritional status of KTx patients requires careful monitoring. Appropriate dietary and lifestyle habits prevent nutrition disturbances and may improve kidney graft function. Despite many nutritional guidelines and recommendations targeted at chronic kidney disease, there are few targeted at KTx recipients. We aimed to provide a brief review of nutrition disturbances and known nutritional recommendations for kidney transplant recipients based on the current literature and dietary trends.

## 1. Etiology of Nutrition Disturbances in Chronic Kidney Disease

Chronic kidney disease (CKD) affects over 10% of the worldwide population and is one of the leading causes of mortality [1]. CKD is inevitably associated with nutrition disturbances. The links between nutritional status and quality of life, psychological well-being, physical fitness, morbidity, or even mortality have been scrutinized for decades. The 2020 National Kidney Foundation’s Kidney Disease Outcomes Quality Initiative (KDOQI) guidelines for nutrition in CKD patients stress the importance of early detection and screening, recommending comprehensive nutritional assessments to be regularly performed [2]. The best treatment of end-stage kidney disease (ESKD) is kidney transplantation (KTx). The nutrition disturbances of CKD patients, including KTx recipients, are complex and depend on the glomerular filtration rate (eGFR), the cause of ESKD, the history and duration of dialysis prior to transplantation, the time since transplantation, pharmacotherapy including immunosuppression both before and after KTx, the comorbidity burden (i.e., diabetes mellitus (DM), including post-transplant DM (PTDM), hypertension, cardiovascular (CV) complications, and bone and mineral disturbances), and finally dietary habits and lifestyle choices [3]. 

Several subtypes of nutrition disturbances have been described in the CKD population. Despite many obese individuals in this population, undernutrition is more common in the forms of protein–energy wasting (PEW), malnutrition-inflammation-atherosclerosis syndrome (MIA syndrome), and dynapenia and uremic wasting [4]; naturally, obese individuals also suffer from these conditions. These pathologies all result from different combinations of chronic inflammation, uremic toxemia, and hypermetabolism [4]. The expert panel gathered by the International Society of Renal Nutrition and Metabolism proposed the following criteria of PEW: reduced overall body (or fat) mass, reduced muscle mass or the presence of sarcopenia, the reduced dietary intake of energy or protein, and low levels of the following serum markers: albumin, cholesterol, and transthyretin (previously known as prealbumin) [4]. Its causes include increased catabolism, dyselectrolytemia, metabolic acidosis, insulin resistance, and other endocrine disorders, such as hyperparathyroidism, hyperglucagonemia, or the deficiency of multiple vitamins including vitamin D. Patients treated with dialysis lose water-soluble micro- and macronutrients during both hemodialysis (HD) and peritoneal dialysis (PD). MIA syndrome describes the coexistence of malnutrition with chronic inflammation and atherosclerosis, all of which are poor prognostic factors for CKD. Reportedly, recipients who exhibit signs of MIA prior to transplantation have a greater risk of post-KTx CV events [3]. Aside from increasing the CV risk, it also impairs muscle function, which leads to dynapenia—a state of decreased muscle strength independent of muscle mass loss [5]. 

The progression of CKD and the associated toxemia exacerbate malnutrition. Diminished appetite is a common complaint of CKD patients, who often suffer from taste disturbances, delayed gastric emptying, and constipation. Uremia and anemia increase the incidences of gastropathy, Helicobacter pylori infection, and uremic gastritis [6]. Bowel edema facilitates bacterial translocation and endotoxemia, the severity of which increase with CKD progression and peak once dialysis is introduced. Through stimulating chronic inflammation, endotoxemia forms a non-traditional CV risk factor in CKD [7]. Metabolic acidosis, which occurs more frequently with CKD progression, contributes to malnutrition via appetite loss, taste distortion, and MIA exacerbation. Correcting acid–base disturbances has the potential of improving malnutrition [8]. The malnutrition burden is characteristic of ESKD patients who then undergo kidney transplantation. 

KTx as the best form of kidney replacement therapy poses different nutritional challenges. A functioning allograft not only relieves uremia, acidosis, and electrolyte disturbances, but also resumes other kidney functions such as erythropoietin production and vitamin D3 metabolism. Studies suggest that kidney transplantation is protective against endotoxemia-induced chronic inflammation associated with bacterial translocation [9]. However, it is important to remember that only a small percentage of KTxs are preemptively performed; most recipients are treated before KTx with maintenance HD or rarely with PD. Moreover, most recipients present with chronic kidney graft disease long-term after KTx, usually from stages G2–G3T [10]. Hence, prior to transplantation, recipients often present with mild to advanced nutrition disturbances [10]; potential recipients rarely present with obesity, since a BMI > 35kg/m^2^ is considered a modifiable exclusion criterion for KTx in most transplantation centers [11]. 

## 2. BMI in KTx Recipients

KTx recipients represent a whole spectrum of undernutrition and obesity. While a higher BMI is rarely related to adverse outcomes in HD patients [12], it appears disadvantageous in KTx patients [13]. In the perioperative period, obesity is associated with poor wound healing, prolonged hospitalization, and surgical complications including lymphoceles and delayed graft function [14]. In the late post-transplant period, obese individuals are more prone to impaired graft function, the presence of proteinuria, PTDM, and atherosclerosis progression, which lead to hypertension and CV complications [13]. The largest increase in body mass occurs within the first 12 months following transplantation [15]. According to a study conducted in a Polish population, only about 35% of KTx recipients present with a normal BMI in a late post-transplant follow-up, 38% are overweight, and as many as 26% are obese. Overall, BMI increased in 65% of subjects and decreased in 24% after KTx. Interestingly, recipients with a normal BMI prior to KTx experienced a greater increase in BMI compared with their overweight peers; the increase in BMI was 0.6 ± 3.5 kg/m^2^ for obese individuals and 1.9 ± 2.4 kg/m^2^ for those with a normal BMI [15]. Weight gain in the first 12 months is associated with HD vintage; the longer the HD vintage, the greater the weight gain [16]. Although obesity remains the main focus in nutrition studies, being underweight has also been linked to poor graft function [17]. A meta-analysis showed that both BMI extremes—being obese and underweight—at the time of KTx were associated with worse survival and graft function [18]. 

## 3. Immunosuppression and the Nutritional Status

The immunosuppressive regimen alone has a disadvantageous effect on the nutritional status. Calcineurin inhibitors (CNIs: tacrolimus (TAC) and cyclosporine A) and m-TOR inhibitors (everolimus and sirolimus) included in the standard immunosuppressive regimen have an unfavorable metabolic profile. They all lead to dyslipidemia, hypertension, and hyperkalemia. CNIs (mainly TAC) and m-TOR inhibitors, as well as glucocorticosteroids (GCSs), may cause PTDM. Additionally, they may be a cause of fluid retention [19]. Cyclosporin A (CsA) also increases the risk of hyperuricemia [20]. All immunosuppressants, especially mycophenolates, also promote gastritis and intestinal erosions [6]. Mycophenolate mofetil has relatively few metabolic side-effects; however, common side-effects include gastrointestinal distress, which contributes to malabsorption and dysbiosis [21]. Reportedly, one of the most common reasons behind mycophenolate mofetil dose reduction (or rarely, discontinuation) is gastrointestinal distress [22]. 

Adverse effects of GCSs also include a loss of skeletal muscle mass, increased appetite, and visceral obesity [23]. Those combined with alleviated dietary restrictions cause post-KTx weight gain, which as body composition analysis shows, is mostly due to an increase in fat tissue mass. GCSs cause an unfavorable shift in body composition, with increased muscle wasting and adiposity. It was shown that early steroid withdrawal is supposedly linked to an increase in lean mass percentage [24]; however, it could increase the risk of graft rejection. 

Drug–food interactions are crucial in the KTx population to maintain the desired blood trough levels of immunosuppressive medications and to avoid rejection or drug toxicity. CNIs are metabolized via the cytochrome P450 and the CYP3A4 and CYP3A5 enzymes. Restricted products include grapefruit and grapefruit juice, pomelo, and spices such as turmeric and ginger [25,26]. Herbal preparations with known interactions with CNIs include cat’s claw (Uncaria tomentosa), devil’s claw (grapple plant, Harpagophytum procumbens), and milk thistle (Silybum marianum). All of these inhibit the CYP3A4 cytochrome and thus increase blood levels of TAC and CsA; St John’s wort on the other hand is a CYP3A4 inductor and thus decreases blood levels of CNIs [27]. Aside from direct metabolic interactions, different food groups alter the adsorption of the drug itself; high-fat meals delay TAC absorption. Hence, it is recommended to eat at least 2–3 h or 1 h after TAC ingestion [28]. Mycophenolate mofetil on the other hand is often taken alongside meals to mitigate its gastrointestinal side-effects without decreasing the AUC of the mycophenolic acid [27].

In the early post-transplant period, symptoms such as nausea, vomiting, diarrhea, and abdominal pain are highly prevalent as medication doses are typically higher to meet target blood trough levels. 

## 4. Dyslipidemia in KTx 

Over 60% of KTx recipients suffer from dyslipidemia, with hyperlipidemia being the most common presentation. The immunosuppressive regimen is the main risk factor of the development of lipid disturbances in this population [29,30,31]. While HDL levels remain comparable or slightly decrease [32], triglyceride-rich VDL and LVDL particles, more prone to oxidation and thus more atherogenic, increase [31]. This disadvantageous shift in lipidemia is largely attributable to immunosuppressive pharmacotherapy. The negative impact of GCSs on carbohydrate metabolism and CV risk, including dyslipidemia, has been well documented [33]. Furthermore, CsA reduces cholesterol elimination associated with bile excretion through inhibiting the mitochondrial steroid 26-hydroxylase [34]. CsA reportedly increases LDL and triglycerides (TGs), while in subjects receiving TAC-based immunosuppressive schemes, dyslipidemia is less frequent; some studies report no relationship between TAC and blood lipid profiles [35]. Nevertheless, patients treated with TAC have a greater risk of developing PTDM [36]. Dyslipidemia remains one of the most common side-effects of m-TOR inhibitors. It presents as an increase of total cholesterol and LDL and TG levels and is observed in most patients treated with these kinds of immunosuppressive drugs [37]. The m-TOR signaling pathway regulates the uptake of lipids into adipose tissue, their breakdown by lipoprotein lipase [38], and the expression of hepatic LDL receptors [39]. It amounts to a 20–30% reduction in lipid storage and a 20% increase in basal lipolysis [38]. On the other hand, it has anti-atherosclerotic properties, such as improving the endothelial function, decreasing the number of macrophages in the atheromatous plaque, and inducing cholesterol efflux from macrophages, thus decreasing lipid accumulation [40]. Due to their antiproliferative effect on smooth muscle cells and their supposed atheromatous plaque stabilization, m-TOR inhibitors found another application in drug-eluting stents [41]. Ultimately, however, the results of large studies regarding m-TOR inhibitors and CV risk remain inconclusive. 

## 5. Post-Transplant Diabetes Mellitus

PTDM is a frequent complication of KTx, affecting about 10–40% of kidney recipients [42]. Risk factors are often divided into two categories: pre- and post-transplantation. Some pre-transplant risk factors are the same as in type 2 DM: obesity with a BMI > 30 kg/m^2^; an age > 40 years; a family history of DM; and Hispanic, African American, or Asian ethnicity. Several conditions, such as cystic fibrosis, polycystic kidney disease, hepatitis C, and CMV virus infection, also increase the odds of PTDM [19,43]. Post-transplant risk factors are largely associated with the immunosuppressive regimen. GCSs increase insulin resistance, hepatic gluconeogenesis, and the overall caloric intake through appetite stimulation [19]. CNIs may contribute to PTDM on many levels, including β-cell toxicity, as calcineurin regulates the survival of human β-cells in the pancreatic islets [44]. While TAC is seemingly preferable in the context of dyslipidemia, clinical observations and many studies have shown a greater incidence of PTDM in subjects treated with TAC [45]. Wissing et al. conducted a prospective study and found that a conversion from TAC to CsA was associated with improved glucose metabolism, and in 34% of cases, a reversal of PTDM [46]. However, there are no strong recommendations for converting PTDM patients from TAC to CsA. Researchers have also stressed the role of pre-existing damage to the β-cells in cases of insulin-resistant or peridiabetic patients, which predisposes them to post-transplantation CNI-induced damage [47]. CNIs also contribute to the insulin resistance of adipose and skeletal muscle tissue [48]. Likewise, m-TOR inhibitors also contribute to PTDM (in animal models), sirolimus-inhibited β-cell proliferation, as well as the production and secretion of insulin [49]. In fact, the inhibition of the m-TOR pathway was suggested as another mechanism through which TAC induced diabetes [50]. There has been speculation about a possible link between intestinal dysbiosis and the development of PTDM [51]. 

There are no nutritional guidelines specifically targeted at patients with PTDM; thus, they must follow the same basic recommendations for all diabetic and CKD patients: limited monosaccharide consumption, a low salt intake, increased fiber consumption, and moderate physical activity performed for 150 min/week [2]. There are few intervention trials aimed at preventing PTDM. Interventions such as dietary counsel and the regular supervision of physical activity appear beneficial against insulin resistance [52]. The 2022 KIDGO guidelines for DM in CKD place nutrition and lifestyle at the base of the pyramid for lowering CV risk [53]. An increased vegetable intake and Mediterranean dietary patterns may prove beneficial in the prevention and management of PTDM; however, based on a 2022 systematic review, high-quality research is needed to confirm these findings [54]. 

## 6. Vitamin Deficiency in KTx

KTx recipients are prone to vitamin D deficiency from insufficient consumption, avoiding sunlight, and finally impaired kidney metabolism [55]. Due to phosphorous intake restrictions, patients with CKD often end up limiting vitamin D as well, meaning that most recipients present with a deficiency prior to KTx [56]. Post-transplantation, in order to reduce the risk of skin malignancies, they are instructed to avoid excessive sun exposure. This lowers the supply of inactive vitamin D metabolites. The kidneys, particularly the proximal tubule cells, facilitate their activation via 1-alpha hydroxylase. The number of active glomeruli drops with CKD progression, while impaired glomerular filtration increases the concentration of fibroblast growth factor-23 (FGF-23), which down-regulates the activity of 1-alpha hydroxylase. Other known inhibitors of the enzyme found in CKD include acidosis [57] and hyperuricemia [58]. 

Vitamin D levels depend on body mass and composition [59]; in the KTx population, higher adiposity was associated with lower blood concentrations of 25(OH)D [55]. This in turn possibly has an impact on the blood lipid profile, as calcifediol concentrations are inversely correlated with LDL and TG levels [60], additionally depriving the patient of the protective, immunomodulatory effect that vitamin D has on graft survival [61]. KTx recipients present with lower vitamin D levels compared with healthy individuals. The recommended intake for KTx recipients is 600 IU/day; experts suggest monitoring blood levels of 25(OH)D, the reference range of which is the same as in the general population, and they recommend cholecalciferol supplementation in the case of its deficiency [2]. 

Vitamin K, mainly K2, is believed to play a protective role for both CV and skeletal systems [62,63]. Most KTx patients have a vitamin K2 insufficiency [64]. The current adequate intakes for vitamin K for the healthy population are based on the median phylloquinone intakes and for adults aged 19 and older are 90 μg/d for females and 120 μg/d for males [60]. 

Despite the important role of vitamin K2 in many physiological processes, there are no separate dietary requirements for its intake [65]. The existing guidelines only refer to the avoidance of vitamin K supplementation in patients treated with classic oral anticoagulants [2], and there are no specific guidelines for the consumption of vitamins K1 and K2 in the KTx population. 

Vitamin B12 and folic acid ought to be supplemented when subjects show clinical signs of deficiency. However, hyperhomocysteinemia associated with CKD is not in itself a recommended indication for folate and/or B12 supplementation [2]. With kidney transplantation, erythropoietin secretion is restored; however, patients are still prone to anemia [66]. Again, there are no specific dietary guidelines for anemic KTx recipients; hence, incases of iron deficiency, patients should increase its dietary intake, for example, by incorporating more meat, legumes, nuts, and vegetables such as broccoli into their diet [67].

## 7. Hyperuricemia

KTx patients are prone to hyperuricemia, which is an important risk factor of CV complications. It has been established that uric acid levels rise alongside decreasing eGFR—from both transplanted and native kidneys—which is the main risk factor of hyperuricemia aside from CsA treatment [68]. Uric acid levels ought to be periodically monitored in all KTx recipients, but especially in those with impaired eGFR or receiving CsA; a low-purine diet ought to be introduced. Key products to avoid include beer, meat, and its by-products, including animal-derived fats, as well as fatty fish and seafood. Dietitians and clinicians often provide patients with detailed tables with the purine load of different products [69].

The high intake of fructose by patients with kidney failure, including KTx patients, leads to increased serum levels of uric acid and TG [70]. KTx recipients tend to lean towards fructose- and cholesterol-rich diets [31]. Fructose is a popular substance used in the food industry, found in most processed foods; thus, when recommending dietary changes for KTx recipients, one should warn against store-bought jams, sweeteners, and processed snacks.

## 8. Macronutrients: Recommendations for KTx

Several studies prospectively analyzed the macronutrient pre- and post-transplantation intake with inconclusive results ranging from no significant changes within the first 6 months [64] to an increased fat intake observed both at the 3rd and 12th month timepoints [71]. A study conducted in Mexico analyzing the dietary compositions in long-term KTx follow-ups showed that the average recipient’s diet consisted of 25% fat, 15% protein, and 55% carbohydrates [72]. Polish KTx recipients in a long-term follow-up often chose energy-dense foods such as sweets and snacks, which provided them with an average of 449 kcal per day. Moreover, saturated fatty acids accounted for over half of their total fat intake. KTx recipients exceeded the general population recommendations of protein, cholesterol, sugar, phosphorus, and sodium, while consuming insufficient amounts of fiber, potassium, and magnesium [73]. Another research group linked nutritional patterns and the associated changes in body composition to gender, steroid doses, delayed graft function, and the occurrence of acute rejection. Females consumed more protein and calories, and thus experienced post-transplant weight gain [74]. However, according to the CORPOS study, the disadvantageous shift in body composition may be mitigated through lifestyle adjustments, such as increased physical activity [75].

KTx recipients have no fixed set of dietary guidelines safe for those related to food–drug interactions. Most restrictions and recommendations result from individual comorbid conditions (DM, CV diseases, and hypertension), metabolic disorders, and of course graft function and the presence of proteinuria.

The daily protein intake varies based on the time after KTx, graft function, and proteinuria.

## 9. Dietary Recommendations in the Early Post-KTx Period

In the first 4 to 6 weeks after transplantation, tissue recovery combined with stress, increased catabolism, and high doses of GCSs lead to protein hypercatabolism. An adequate protein supply is necessary for quick recovery, wound healing, and lesser susceptibility to infections. Therefore, the early post-KTx period revolves around recovery. The target daily protein intake ranges from 1.2 to 2 g/kg of the ideal body weight [71,76]. As weight loss is not the focus, the caloric intake should fall between 30 and 35 kcal/kilogram of body mass/day [76], 50–70% of that obtained from carbohydrates.

Due to low tubular reabsorption and disproportionately high parathormone levels, hypophosphatemia is common in the first few weeks following KTx [77]. In the early post-KTx period, it is necessary to monitor phosphorus blood levels on a weekly basis, especially in patients with rapidly improving graft function, as they may require high-phosphate products or even their oral supplementation [78,79]. In the early post-transplant period, there is a tendency towards either hypo- or hyperkaliemia. Hyperkalemia is usually a side-effect of medications; 5 to 40% of patients treated with CNIs develop hyperkalemia [79]; other medications include i. a. sulfamethoxazole with trimethoprim, β-blockers, and heparin. If hyperkalemia occurs (mainly in patients with impaired graft function), the potassium intake should be reduced to 3 g/day [80], once other reversible causes of hyperkalemia such as metabolic acidosis, which is common in the early post-KTx period, have been excluded. Without surgical contraindications, oral nutrition including solid meals may be introduced 2–3 days following the procedure. Enteral or parenteral nutrition should be considered if nausea, ileus, or persistent vomiting prevent oral nutrition for more than 5 days [2].

Gastrointestinal distress, dyspepsia, and diarrhea are often observed in the early weeks after transplantation. These often result from immunosuppressive medications, mainly mycophenolate mofetil and TAC. Thus, from our clinical practice, products including or based on milk, as well as rich in fiber, should be excluded to avoid the further exacerbation of gastrointestinal distress. Instead, an easily digestible diet is preferred. In addition, as hyperglycemia is often observed, monosaccharide intake is restricted.

Recipients’ physical fitness in the early post-KTx phase is limited by their low activity level from the dialysis period, early post-operation stage and frequently suboptimal allograft function, anemia, fluid overload, and mineral disturbances [81,82]. As soon as there are no contraindications, patients ought to perform moderate exercise for at least 30 min, five times a week [83].

## 10. Dietary Recommendations in the Long Term after KTx

Dietary recommendations for recipients in the long term after KTx depend largely on kidney graft function, maintenance therapy, including immunosuppressive drugs, and pre-existing and new comorbidities, as well as PTDM, HA, CV diseases, lipid disturbances, or hyperuricemia. Patients with good and stable graft function ought to follow the same basic recommendations as the general population. Likewise, they can partake in the same physical activity recommended for their age and non-renal comorbidity burden [84,85].

A prospective study showed that the physical activity of KTx recipients increased up to 30% and reached a plateau after the first 12 months of follow-up [85]. The KDOQI recommends moderate-intensity physical activity to be performed five times a week for 30 min. Aside from physical health benefits, exercise interventions potentially improve quality of life [86]. They also positively impact the lipid profile, particularly HDL levels [81]. In these times, patients should be encouraged to take advantage of mobile applications and wearable gear dedicated to fitness in order to track progress and increase mental motivation.

During the maintenance period, the recommended daily energy intake for KTx patients should be 25–35 kcal/kg/day [2]; adjustments ought to be made accordingly for under- or overweight patients. Unlike in the first 4–6 weeks post-KTx, about 45–50% of the daily caloric intake should come from carbohydrates [76]. The 2020 KDOQI guidelines do not specify the target protein intake for KTx recipients, only referring to CKD and ESKD populations. According to other sources, the estimated protein intake would be 0.6–0.8 g/kg/day in the case of non-diabetic and 0.8–0.9 g/kg/day in the case of diabetic patients [76]. Other sources indicate that KTx recipients should not exceed 0.75 g/kg/d for females and 0.84 g/kg/d for males to maintain good graft function and overall wellbeing [87]. Per KDOQI, we lack sufficient data to proclaim if either plant- or animal-based sources of protein are superior and thus preferred [2]. Patients with preexisting diabetes or those who develop PTDM should rather choose complex carbohydrates over monosaccharides and maintain a high fiber intake. The daily fiber intake should be 25–35 g per day [88]; this also helps prevent constipation and thus hyperkalemia, bacterial translocation, and diverticulitis.

Due to a high prevalence and risk of dyslipidemia, KTx recipients are recommended to follow a low-fat, low-cholesterol diet. Between 30 and 35% of calories consumed daily should come from fats with less than 8–10% coming from polyunsaturated and trans fatty acids, while some sources suggest that monounsaturated fatty acids may account for up to 20% of daily calories [29]. Additionally, a diet high in fiber and low in trans fats helps maintain normal blood glucose levels and lower TG and LDL cholesterol. KDOQI guidelines suggest prescribing the Mediterranean diet to improve the lipid profile.

As hypertension arteriosum is highly prevalent in the KTx population, over 90% of recipients require anti-hypertensive medication [89]; HA necessitates a low-sodium diet, the recommended intake of which per KDOQI guidelines is 2–3 g/day [2].

## 11. Dietary Patterns in the KTx Population

While recommendations individually refer to macro- and micronutrients, patients consume their meals as a whole, and thus, while specific guidelines prove useful for clinicians, individual patients require a more realistic, comprehensible approach, such as whole dietary patterns.

The Dietary Approaches to Stop Hypertension (DASH) has been investigated as a potentially beneficial intervention in KTx due to its proven effect on high blood pressure. The key principles include a low sodium intake and the moderate consumption of lean protein and fish with the avoidance of red and processed meats, combined with a high intake of fruits, vegetables, whole grains, low-fat dairy, and fiber. This pattern facilitates low fat consumption, with a preference of monounsaturated fats over saturated and trans fats. In a large cohort study of over 600 KTx recipients, the DASH eating pattern was associated with a lower risk of decreased graft function and all-cause mortality [90]. In the general population, the DASH diet significantly improves blood pressure, total cholesterol, and LDL serum concentrations.

Likewise, the Mediterranean diet has a proven beneficial effect on kidney graft function [91]. It focuses on planning meals rich in whole grains, vegetables, fruits, seeds, nuts, beans, legumes, and olive oil, with fish consumed on a twice-a-week basis. The predominance of unsaturated fats over saturated fats found in red meat reduces oxidative stress, chronic inflammation, and atherosclerosis [92,93]. Vučković et al. explored the links between sticking to a Mediterranean diet, body composition, and depression symptoms and found an association between low muscle mass and depression symptoms [94].

Both of these eating patterns appear beneficial against insulin resistance, inflammation, oxidative stress, and dyslipidemia [93]. Interestingly enough, the 2020 KDQOI guidelines mention only the Mediterranean diet as a potential means of improving lipid profiles.

In recent years, the interest in vegetarian and vegan diets has increased worldwide. The potential superiority of plant-based sources of protein has spiked an ongoing debate in the nephrology community [95]. Plant-based diets help alleviate acidosis and prevent hyperphosphatemia, as the phosphorus derived from plants is more difficult to absorb into the gastrointestinal tract. Moreover, they have a higher fiber content, which is necessary to maintain a healthy gut microbiome. High-fiber diets help decrease production of uremic toxins associated with the microbiome and lower the risk of obesity, diabetes, and dyslipidemia. However, patients who follow vegetarian diets are at a higher risk of iron-deficiency anemia; dairy and eggs remain the only source of vitamin B12; in addition, plant-based iron has lower bioavailability [96]. Owing to multiple drug–food interactions, monitoring immunosuppressant trough levels is crucial during major dietary changes.

Due to the high incidence of mineral and bone disorders in this population, recipients must monitor their calcium intake; the recommended daily dose is 800–1000 mg, unless hypercalcemia occurs [2], with a phosphorus intake of 1200–1500 mg/day [71,83].

## 12. Nutritional Screening

The key to preventing malnutrition is screening; bi-annual assessments are recommended to find patients at risk of developing both PEW and obesity [2]. In KTx recipients, DXA remains the gold standard for body composition analysis; however, skinfold calipers suffice for body fat measurements in individuals without edema. According to experts, it may prove useful to assess body composition alongside classic measurements such as body mass and BMI at the first appointment and periodically monitor them every 3 months in the case of KTx recipients [2]. In addition, the Malnutrition Inflammation Score may be applied; serum biomarkers such as albumin and transthyretin may serve as complementary tools.

## 13. Conclusions

Preventing nutrition disturbances is crucial in the KTx population in order to minimize the risk of CV events, metabolic complications, and to maintain good graft function. A comprehensive set of guidelines dedicated to kidney transplant recipients ought to be developed to aid physicians and clinical dietitians in providing the patients with the best care possible.

## Data Availability

Not applicable.
